# Effect of Planar Interfaces on Nucleation in Melting and Crystallization

**DOI:** 10.3390/e24081029

**Published:** 2022-07-26

**Authors:** Jürn W. P. Schmelzer, Azat O. Tipeev

**Affiliations:** 1 Institut für Physik, Universität Rostock, Albert-Einstein-Strasse 23-25, 18059 Rostock, Germany; 2 Competence Centre CALOR, Faculty of Interdisciplinary Research, University of Rostock, Albert-Einstein-Str. 25, 18051 Rostock, Germany; 3 Institute of Metallurgy, Ural Branch of the Russian Academy of Sciences, 620016 Ekaterinburg, Russia; azattipeev@gmail.com; 4 Institute for High Pressure Physics, Russian Academy of Sciences, 108840 Troitsk, Moscow, Russia

**Keywords:** nucleation, thermodynamics of nucleation, general theory of phase transitions, crystallization, melting, 64.60.Bd General theory of phase transitions, 64.60.Q Nucleation, 81.10.Aj Theory and models of crystal growth, 64.70.D Solid–liquid transitions, 82.60.Nh Thermodynamics of nucleation in physical chemistry and chemical physics

## Abstract

The effect of planar interfaces on nucleation (namely, on the work of critical cluster formation and their shape) is studied both for crystallization and melting. Advancing an approach formulated about 150 years ago by J. W. Gibbs for liquid phase formation at planar liquid–liquid interfaces, we show that nucleation of liquids in the crystal at crystal–vapor planar interfaces proceeds as a rule with a much higher rate compared to nucleation in the bulk of the crystal. Provided the surface tensions crystal–liquid ($\sigma_{cl}$), liquid–vapor ($\sigma_{lv}$), and crystal–vapor ($\sigma_{cv}$) obey the condition $\sigma_{cv}=\sigma_{cl}+\sigma_{lv}$, the work of critical cluster formation tends to zero; in the range $\sigma_{cv}<\sigma_{cl}+\sigma_{lv}$, it is less than one half of the work of critical cluster formation for bulk nucleation. The existence of a liquid–vapor planar interface modifies the work of critical cluster formation in crystal nucleation in liquids to a much less significant degree. The work of critical crystal cluster formation is larger than one half of the bulk value of the work of critical cluster formation, reaching this limit at $\sigma_{cv}=\sigma_{cl}+\sigma_{lv}$. The shape of the critical clusters can be described in both cases by spherical caps with a radius, *R*, and a width parameter, *h*. This parameter, *h*, is the distance from the cutting plane (coinciding with the crystal–vapor and liquid–vapor planar interface, respectively) to the top of the spherical cap. It varies for nucleation of a liquid in a crystal in the range (h/R)≤1 and for crystal nucleation in a liquid in the range 2≥(h/R)≥1. At $\sigma_{cv}=\sigma_{cl}+\sigma_{lv}$, the ratio (h/R) of the critical cluster for nucleation in melting tends to zero ((h/R)→0). At the same condition, the critical crystallite has the shape of a sphere located tangentially to the liquid–vapor interface inside the liquid ((h/R)≅2). We present experimental data which confirm the results of the theoretical analysis, and potential further developments of the theoretical approach developed here are anticipated.

## 1. Introduction

In order to formulate the basic theoretical concepts governing nucleation and growth of a new phase in a metastable ambient phase, homogeneous nucleation in the bulk of the metastable initial phases is commonly analyzed as a first step [1,2,3,4,5,6,7]. This analysis is supplemented by the account of the effect of heterogeneous nucleation sites dissolved in the bulk of the ambient phase on nucleation. Heterogenous nucleation sites may be located at the surfaces of the homogeneous initial phases as well, and considerably affect the nucleation and growth processes. Examples in this respect are the dependence of crystal nucleation on prior liquid overheating and the commonly observed preferential surface crystallization of glasses [8,9,10].

With respect to the latter observation, it has been shown that the inhibiting nucleation effect of elastic stresses is much more expressed in the bulk as compared to the interface of a glass or highly viscous liquid. In this way, the higher rate of crystal nucleation near interfaces can be explained, exclusively accounting for the interplay of stress evolution and relaxation in crystallization [11,12]. Specific properties of surface crystallization have been observed in other applications [13,14,15,16] and connected with particular features of the structure of the liquid near to the surface. In the present analysis, we neglect heterogenous nucleation sites, specific features of the structure of the ambient phase and the effect of elastic stresses. Instead we analyze the problem of whether the mere existence of a liquid–vapor planar interface affects the rate of crystal nucleation processes in liquids.

A similar problem concerning the dependence of the rate of nucleation processes on the existence of a planar interface has been observed in the analysis of melting processes. As noted long ago by A. Ubbelohde [17,18,19], *“a crystal cannot be readily superheated implies that this rate process proceeds rapidly and without difficulty at the surface of a solid”*. Indeed, in contrast to supercooling and superheating of a liquid, the superheating of a solid is very difficult to achieve. Nonetheless, a solid with an open surface can be superheated in particular experiments at appropriate conditions [20,21,22,23,24,25,26,27,28,29,30,31], for example, during rapid heating of a solid throughout its volume and simultaneous cooling of its surface to suppress surface melting [20,30]. Superheating can be obtained in tiny crystalline clusters inserted into a proper medium with a higher melting temperature by their subsequent laser heating [22,25,26,29]. Depending on the coating material, either volume or surface nucleation might be favored [21]. Finally, considerable superheating can be achieved via ultra-fast shockwave compression of solids [27,28]. However, the problem remains as to why melting near interfaces is obviously significantly favored compared to nucleation of a liquid aggregate in the bulk of a solid. The answer to this question is the second main topic of the analysis presented in this paper.

Despite significant technological progress and the variety of studies devoted to it, the mechanisms of surface nucleation are not fully understood. Considering the aforementioned arguments, the main objective of the current study is thus to develop a theoretical thermodynamic model of nucleation at crystal–vapor and liquid–vapor planar interfaces and provide insight into the extent to which surface nucleation is favored over the bulk nucleation in terms of classical nucleation theory (CNT). As CNT is known to correctly describe the basic features of nucleation independent of the particular application, it can be expected to supply us with an adequate insight into the effect of planar interfaces on nucleation and its differences for nucleation in crystallization and melting. Of course, depending on the particular case studied other effects may have to be incorporated into the description in order to arrive at a quantitatively correct treatment. However, such possible particular features and their effects on nucleation are beyond the scope of the present analysis.

**Figure 1:**
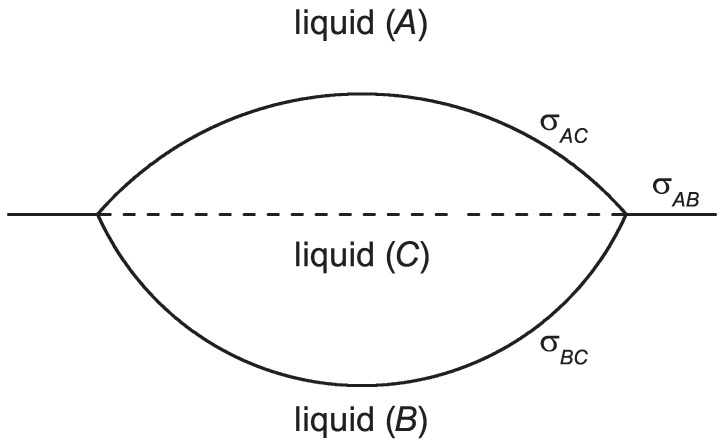
Model of the description of nucleation of a new liquid phase at a planar liquid–liquid interface, as analyzed by J. W. Gibbs [32]. In his approach, the critical cluster is described as being formed of two spherical caps with radii $R_{1}$ and $R_{2}$; $\sigma_{AB}$, $\sigma_{BC}$, and $\sigma_{AC}$ denote the respective values of the surface tension.

In order to arrive at an answer to the questions formulated above, we advance here an approach first developed by J. W. Gibbs in the section “*On the Possible Formation at the Surface where two different Homogeneous Fluids meet of a Fluid of different Phase from either*” of his fundamental treatment [32]. His model is illustrated in Figure 1. According to his approach, at the planar interface between two different liquids, A and B, a region containing a new liquid phase, C, is formed. This region, as noted by Gibbs, is unstable and will grow further once it has been developed; it has thus the meaning of a critical cluster as discussed in nucleation theory. The work of forming this region was considered by Gibbs as a measure of stability of the liquid–liquid coexistence of liquids A and B. In particular, Gibbs arrived at the conclusion that the work of formation of such aggregates may tend to zero if the condition $\sigma_{AC}+\sigma_{BC}=\sigma_{AB}$ holds. One of the aims of our analysis is to check whether such condition may hold at nucleation at liquid–vapor or solid–vapor planar interfaces.

Models of a similar type have been analyzed in subsequent investigations in different directions, such as condensation and boiling or segregation in solutions at planar solid interfaces [1,33,34], wetting of solids by liquids, i.e., the ability of a liquid to maintain contact with a solid surface [35,36,37], bubble nucleation in liquids [38], and in the above-mentioned analysis of the effect of elastic stresses on crystal nucleation [9,10,11,12]. To the best of our knowledge, as presented here it is utilized for the comparative analysis of the effects of planar interfaces on nucleation in melting and crystallization for the first time.

The paper is structured as followed. In Section 2.1, we describe the model employed for the description of nucleation in the bulk and then apply it to the analysis of nucleation near planar interfaces in melting of crystals (Section 2.2) and crystallization of liquids (Section 2.3). In both cases, we determine the work of critical cluster formation and the shape of the critical clusters and compare them with the respective quantities obtained for bulk nucleation. A summary of the results and their discussion (Section 3) completes the paper.

## 2. Theoretical Analysis of Nucleation Near Planar Interfaces

### 2.1. The Model: Nucleation in the Bulk

In the present analysis, we concentrate our attention on the melting of crystals, respectively, the crystallization of liquids. The processes are assumed to proceed at fixed values of pressure, *p*, and temperature, *T*. At such boundary conditions, the work of critical cluster formation, $W_{c}$, is equal to the change in the Gibbs free energy, $\Delta G_{c}^{\left(cluster\right)}$, caused by the formation of liquid drops or crystallites of critical sizes. Following the suggestions of the founders of classical nucleation theory (see, e.g., [1,2,3,4,7]) employing in their analysis the thermodynamic treatment developed by Gibbs [32], the work of critical crystal cluster formation for spherical critical clusters and the expression for their radius is commonly written as

(1) $$W_{c}=\frac{16\pi }{3}\frac{\sigma_{cl}^{3}}{{\left(\Delta g_{df}^{\left(bulk\right)}\left(T,p\right)\right)}^{2}}\;,\;R_{c}=\frac{2\sigma_{cl}}{\Delta g_{df}^{\left(bulk\right)}\left(T,p\right)}\;,$$

where $R_{c}$ is the radius of the critical cluster. Here, $\Delta g_{df}^{\left(bulk\right)}\left(T,p\right)$ (denoted as the thermodynamic driving force (df) of nucleation and growth) is the difference between the bulk Gibbs free energies per unit volume of the crystal and the melt, both taken at the same pressure *p* and temperature *T*, and $\sigma_{cl}$ is the surface tension for phase coexistence of crystal and liquid.

As a rule, at certain reasonable approximations these relations may be obtained from the expression

(2) $$\Delta G^{\left(cluster\right)}=-\Delta g_{df}^{\left(bulk\right)}\left(T,p\right)V+\sigma_{cl}A\;.$$

Here *V* is the volume and *A* the surface area of a cluster of the newly evolving phase. For simplicity of notation, we replace $\Delta g_{df}^{\left(bulk\right)}\left(T,p\right)$ in our further computations with Δg. As shown in [39,40,41], with the assumptions commonly employed in CNT this relation holds for multi-component systems. In addition, it can be shown that the formation of crystallites can be also appropriately modeled describing their size via the radius, *R*, i.e., $V=\left(4\pi /3\right)R^{3}$ and $A=4\pi R^{2}$.

Employing a relation similar to Equation (2), we now analyze nucleation at planar interfaces, first formulating the expressions for the work of cluster formation for arbitrary values of the chosen parameters describing their shape and size, and afterwards determining their values for the respective equilibrium states corresponding to the critical cluster. The shape of the critical clusters are described in line with the approach followed by Gibbs. In both cases considered by us we are using spherical caps with a radius *R*. The second parameter is provided by the width parameter *h*, the distance from the cutting plane to the top of the spherical cap. The cutting plane coincides with the crystal–vapor and liquid–vapor planar interfaces, correspondingly.

### 2.2. Nucleation in Melting

**Figure 2:**
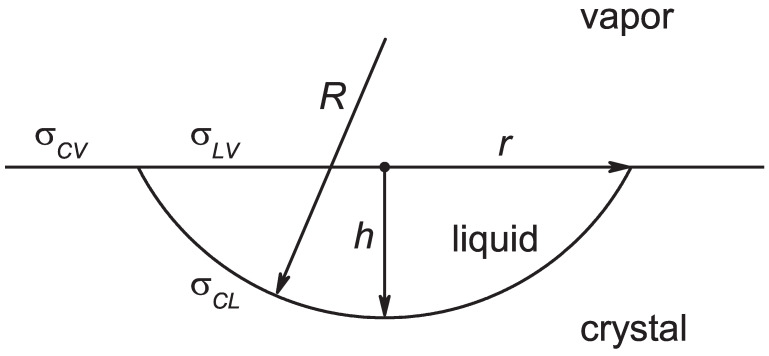
Model for the description of nucleation at planar interfaces in melting.

We first compare the above given result, Equation (1), for the shape of the critical clusters and the work of critical cluster formation formed in the bulk of the crystal for the case in which nucleation proceeds at the crystal–vapor planar interface. The notations employed for the description of the evolving aggregates of the new phase are illustrated in Figure 2.

The work of formation of an aggregate of a liquid with a shape provided by the parameters *R*, *r*, and *h* can be expressed in terms of CNT similarly to Equation (2), as follows:

(3) $$\Delta G^{\left(cluster\right)}=-\Delta gV_{l}+\sigma_{cl}A_{cl}+\left(\sigma_{lv}-\sigma_{cv}\right)A_{lv}$$

with [42]

(4) $$V_{l}=\frac{\pi h^{2}}{3}\left(3R-h\right)\;,\;A_{cl}=2\pi Rh\;,\;A_{lv}=\pi r^{2}\;.$$

Here, the surface tensions for liquid–vapor ($\sigma_{lv}$), crystal–vapor ($\sigma_{cv}$), and crystal–liquid ($\sigma_{cl}$) coexistence are employed. The three shape parameters *R*, *r*, and *h* obey the relation

(5) $$R=\frac{r^{2}+h^{2}}{2h}\;.$$

Utilizing the above, we can rewrite Equation (3) as

(6) $$\Delta G^{\left(cluster\right)}=-\Delta g\frac{\pi h^{2}}{3}\left(3R-h\right)+2\pi Rh\sigma_{cl}+\pi \left(2hR-h^{2}\right)\left(\sigma_{lv}-\sigma_{cv}\right)\;.$$

The critical cluster size is provided by

(7) $$\frac{\partial \Delta G^{\left(cluster\right)}}{\partial R}=\pi h\left\{-\Delta gh+2\left(\sigma_{cl}+\sigma_{lv}-\sigma_{cv}\right)\right\}=0$$

or

(8) $$h_{c}=\frac{2}{\Delta g}\left(\sigma_{cl}+\sigma_{lv}-\sigma_{cv}\right)$$

and

(9) $$\frac{\partial \Delta G^{\left(cluster\right)}}{\partial h}=2\pi R\left\{-h\Delta g+\left(\sigma_{cl}+\sigma_{lv}-\sigma_{cv}\right)\right\}+\pi h\left\{h\Delta g-2(\sigma_{lv}-\sigma_{cv})\right\}=0\;,$$

which along with Equation (8) result in

(10) $$R_{c}=h_{c}\frac{\sigma_{cl}}{\sigma_{cl}+\sigma_{lv}-\sigma_{cv}}\;,$$

respectively,

(11) $$R_{c}=\frac{2\sigma_{cl}}{\Delta g}\;.$$

The radius, $R_{c}$, is consequently determined by the same relation as the critical cluster radius in bulk crystallization, provided by Equation (1). Employing Equations (6), (8), (10), and (11), the work of critical cluster formation, $\Delta G_{c}^{\left(cluster\right)}$, can be written as

(12) $$\Delta G_{c}^{\left(cluster\right)}=\pi R^{2}\sigma_{cl}\left\{-\frac{1}{3}{\left(\frac{h_{c}}{R_{c}}\right)}^{3}+{\left(\frac{h_{c}}{R_{c}}\right)}^{2}\right\}\;,$$

where the ratio $\left(h_{c}/R_{c}\right)$ is provided by

(13) $$\frac{h_{c}}{R_{c}}=\frac{\sigma_{cl}+\sigma_{lv}-\sigma_{cv}}{\sigma_{cl}}\;.$$

According to the model illustrated in Figure 2, both *R* and *h* have to be positive quantities. By this reason, the present model is applicable for systems obeying the condition

(14) $$\sigma_{cv}\leq \sigma_{cl}+\sigma_{lv}\;.$$

**Figure 3:**
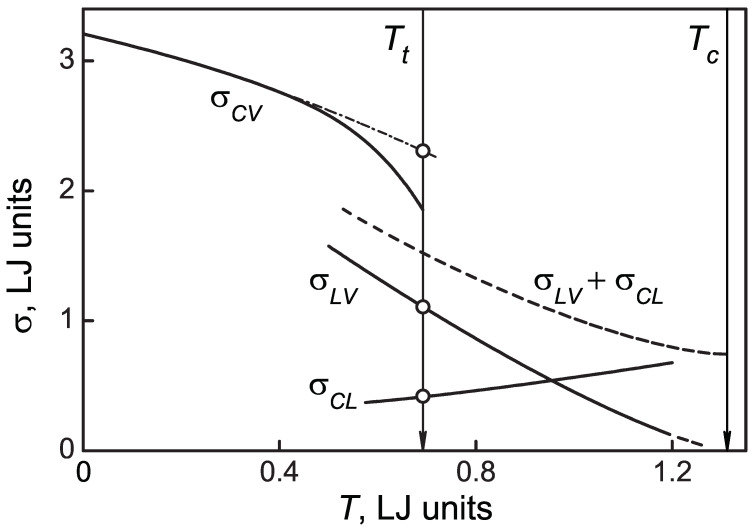
Results of molecular dynamics simulations: temperature dependence of the surface tensions $\sigma_{lv}\left(T\right)$ [45], $\sigma_{cl}\left(T\right)$ [46], and $\sigma_{cv}\left(T\right)$ [47,48]; $T_{t}$ and $T_{c}$ are the triple and critical point temperatures, respectively. The circles at $T=T_{t}$ correspond to values of surface tensions at the triple point. The units are in dimensionless Lennard–Jones form.

Accounting for the Stefan–Skapski–Turnbull rule [3,4,43], the inequality

(15) $$\sigma_{lv}<\sigma_{cv}$$

can be expected to be fulfilled. Indeed, according to this rule, the surface tension is proportional to the specific heat or enthalpy, ΔH, of the phase transformation considered, i.e., σ∝ΔH. Accounting for $\Delta H_{lv}<\Delta H_{cv}$, we arrive at the mentioned inequality. This conclusion is reconfirmed by the molecular dynamics computation results shown in Figure 3. With this condition and Equation (14), Equation (13) results in $0\leq (h_{c}/R_{c})\leq 1$.

In the considered interval, $0\leq (h_{c}/R_{c})\leq 1$, the work of critical cluster formation, Equation (12), is a monotonously increasing function of the ratio $\left(h_{c}/R_{c}\right)$. Assuming strong validity of the Stefan–Skapski–Turnbull rule, with $\Delta H_{cv}=\Delta H_{cl}+\Delta H_{lv}$ we arrive at $\sigma_{cv}≅\sigma_{cl}+\sigma_{lv}$. The validity of the relation $\sigma_{cv}≅\sigma_{cl}+\sigma_{lv}$ for a variety of systems was stressed by Skripov and Faizullin as well in their analysis of similarities and differences in solid–liquid and liquid–vapor phase transitions [44]. At this condition, we obtain $(h_{c}/R_{c})\to 0$ and the work of critical cluster formation tends to zero, i.e., in nucleation near interfaces in melting a similar behavior can be found as that discussed by Gibbs in his analysis of the formation of a new liquid phase in between two liquids. In the alternative limiting case realized at $\left(\sigma_{lv}-\sigma_{cv}\right)\ll \sigma_{cl}$ we arrive at $h_{c}=R_{c}$, and the work of formation of the critical cluster is equal to $\Delta G_{c}^{\left(cluster\right)}=\left(2\pi /3\right)R^{2}\sigma_{cl}$. This is less than the respective value in the bulk, $\Delta G_{c}^{\left(cluster\right)}=\left(4\pi /3\right)R^{2}\sigma_{cl}$, by a factor one half.

Generally, we reach the conclusion that nucleation of critical clusters of the liquid proceeds at a much higher rate near a planar interface of the crystal compared to nucleation in the bulk. As a consequence homogeneous nucleation in melting is significantly enhanced by the existence of planar interfaces.

The analysis performed here is applicable to systems obeying the inequality given by Equation (14). In addition, it is assumed, in line with the Stefan–Skapski–Turnbull rule, that the relation given by Equation (15) always holds. We now briefly test the general validity of these relations based on the results of computer simulations. To the best of our knowledge, the complete set of surface tensions, $\sigma_{lv}\left(T\right)$, $\sigma_{cl}\left(T\right)$, and $\sigma_{cv}\left(T\right)$, required for the analysis of the problems under consideration have been obtained so far for only one substance described by a Lennard–Jones model [45,46,47,48]. The results are presented in Figure 3. In the figure, surface tensions are provided in Lennard–Jones units. At the triple point ($T=T_{t}$), $\sigma_{lv}=1.108$ was obtained based on the analysis of the pressure tensor components [45], $\sigma_{cl}=0.420$ was computed via a cleaving wall method [46], and $\sigma_{cv}=2.308$ was determined via thermodynamic integration [47] and corrected by a recently developed computational crystal cleavage approach [48]. The values of $\sigma_{cv}$ and $\sigma_{cl}$ were averaged over (100), (110), and (111) crystallographic planes.

**Figure 4:**
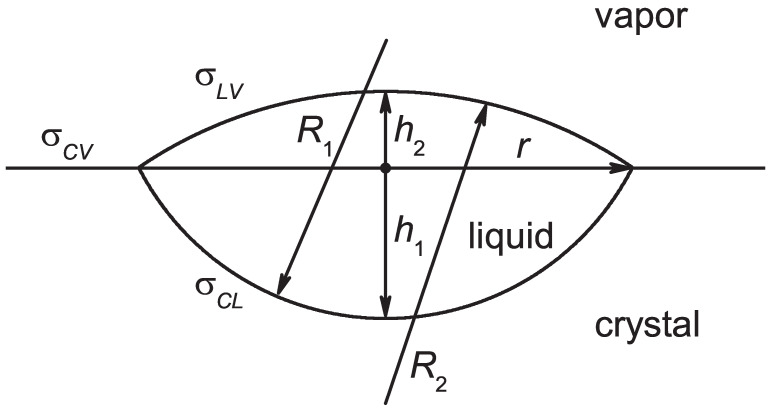
Extension of the model for the description of nucleation of a new liquid phase at a planar liquid–solid interface. In this extension of the model illustrated in Figure 2, the critical cluster is described as being formed of two spherical caps with the radii $R_{1}$ and $R_{2}$ and the height parameters $h_{1}$ and $h_{2}$.

As is evident from Figure 3, the inequality given by Equation (15) is fulfilled in line with our expectations, while the inequality given by Equation (14) is not. In order to describe the behavior of systems not obeying the latter relation, it may be necessary to extend our model. As one such straightforward extension, a shape could be assumed, as suggested by J. W. Gibbs in his analysis of phase formation near liquid–liquid planar interfaces (see Figure 1). An example is shown in Figure 4. Instead of Equation (6), in this case we obtain the following relation for the work of cluster formation:

(16) $$\Delta G^{\left(cluster\right)}=-\Delta g\left\{\frac{\pi h_{1}^{2}}{3}\left(3R_{1}-h_{1}\right)+\frac{\pi h_{2}^{2}}{3}\left(3R_{2}-h_{2}\right)\right\}$$

$$+2\pi R_{1}h_{1}\sigma_{cl}+2\pi R_{2}h_{2}\sigma_{lv}-\pi \left(2h_{1}R_{1}-h_{1}^{2}\right)\sigma_{cv}\;.$$

In line with Equation (5), the relation

(17) $$2R_{1}h_{1}-h_{1}^{2}=2R_{2}h_{2}-h_{2}^{2}$$

holds. Consequently, only three of the four parameters, $R_{1}$, $h_{1}$, $R_{2}$, and $h_{2}$, are independent.

In computing the parameters of the critical clusters for this model we assume that $R_{2}=R_{2}\left(R_{1},h_{1},h_{2}\right)$, i.e., that $R_{2}$ is uniquely determined by the other three parameters. The first of the equilibrium conditions can then be written in the form

(18) $$\frac{\partial \Delta G^{\left(cluster\right)}}{\partial R_{1}}={\left.\frac{\partial \Delta G^{\left(cluster\right)}}{\partial R_{1}}\right|}_{h_{1},h_{2},R_{2}}+{\left.\frac{\partial \Delta G^{\left(cluster\right)}}{\partial R_{2}}\right|}_{h_{1},h_{2},R_{1}}{\left.\frac{\partial R_{2}}{\partial R_{1}}\right|}_{h_{1},h_{2}}=0\;.$$

This condition leads to similar results as Equation (8), to

(19) $$h_{1c}+h_{2c}=\frac{2}{\Delta g}\left[\sigma_{cl}+\sigma_{lv}-\sigma_{cv}\right]\;.$$

The second equilibrium condition,

(20) $$\frac{\partial \Delta G^{\left(cluster\right)}}{\partial h_{1}}={\left.\frac{\partial \Delta G^{\left(cluster\right)}}{\partial h_{1}}\right|}_{R_{1},h_{2},R_{2}}+{\left.\frac{\partial \Delta G^{\left(cluster\right)}}{\partial R_{2}}\right|}_{h_{1},h_{2},R_{1}}{\left.\frac{\partial R_{2}}{\partial h_{1}}\right|}_{R_{1},h_{2}}=0\;,$$

yields

(21) $$R_{1c}=\frac{2\sigma_{cl}}{\Delta g}\;.$$

The third equilibrium condition,

(22) $$\frac{\partial \Delta G^{\left(cluster\right)}}{\partial h_{2}}={\left.\frac{\partial \Delta G^{\left(cluster\right)}}{\partial h_{2}}\right|}_{R_{1},h_{1},R_{2}}+{\left.\frac{\partial \Delta G^{\left(cluster\right)}}{\partial R_{2}}\right|}_{h_{1},h_{2},R_{1}}{\left.\frac{\partial R_{2}}{\partial h_{2}}\right|}_{R_{1},h_{1}}=0\;,$$

results in

(23) $$R_{2c}=\frac{2\sigma_{lv}}{\Delta g}\;.$$

Equations (17), (19), (21), and (23) allow us a complete determination of the parameters of the critical clusters. Similarly to the previously discussed case, they are physically reasonable only if the condition given by Equation (14) is fulfilled. Substitution of these parameters into Equation (16) again supplies us with the value of the work of critical cluster formation. Qualitatively, the results are the same as discussed in the analysis of the first model. In particular, provided that the relation $\sigma_{cl}+\sigma_{lv}-\sigma_{cv}≅0$ holds, the work of critical cluster formation again tends to zero.

We thus arrive at the conclusion that also this more general approach does not describe the shapes of critical clusters when the condition given by Equation (14) is not fulfilled. Which shapes could eventually lead to physically reasonable results in such cases is a question we consider an open problem. One alternative approach to its resolution is discussed in Section 3.

### 2.3. Nucleation in Crystallization

**Figure 5:**
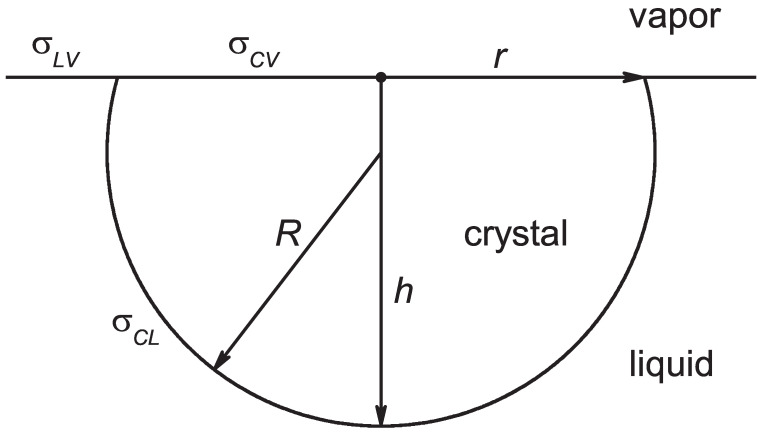
Model for the description of nucleation at planar interfaces in crystallization.

Employing the same model as utilized for the analysis of nucleation in melting to describe crystal nucleation at a liquid–vapor planar interface (see Figure 5), we obtain instead of Equation (6)

(24) $$\Delta G^{\left(cluster\right)}=-\Delta g\frac{\pi h^{2}}{3}\left(3R-h\right)+2\pi Rh\sigma_{cl}+\pi \left(2hR-h^{2}\right)\left(\sigma_{cv}-\sigma_{lv}\right)\;.$$

In this case, the parameters of the critical cluster are determined similarly to Equations (8), (10), and (11) via

(25) $$h_{c}=\frac{2}{\Delta g}\left[\sigma_{cl}+\left(\sigma_{cv}-\sigma_{lv}\right)\right]\;,$$

(26) $$R_{c}=h_{c}\frac{\sigma_{cl}}{\sigma_{cl}+\left(\sigma_{cv}-\sigma_{lv}\right)}\;.$$

As evident from Equations (25) and (26), $R_{c}$ can be expressed as

(27) $$R_{c}=\frac{2\sigma_{cl}}{\Delta g}\;.$$

The work of critical cluster formation is provided in this case via

(28) $$\Delta G_{c}^{\left(cluster\right)}=\pi R^{2}\sigma_{cl}\left\{-\frac{1}{3}{\left(\frac{h_{c}}{R_{c}}\right)}^{3}+{\left(\frac{h_{c}}{R_{c}}\right)}^{2}\right\}\;,$$

again, where the ratio $\left(h_{c}/R_{c}\right)$ can now be written in the following form

(29) $$\frac{h_{c}}{R_{c}}=\frac{\sigma_{cl}+\left(\sigma_{cv}-\sigma_{lv}\right)}{\sigma_{cl}}\;.$$

Accounting again for the generally valid condition $\sigma_{cv}-\sigma_{lv}>0$ (Equation (15)), we arrive at $(h_{c}/R_{c})>1$. In the range $1\leq (h_{c}/R_{c})\leq 2$, the respective value of the work of critical cluster formation is smaller compared to nucleation in the bulk, although the effect is much less significant compared to melting. Vanishing of the work of critical cluster formation under certain conditions is excluded. Assuming strong validity of the Stefan–Skapski–Turnbull rule, with the limit $\sigma_{cv}≅\sigma_{cl}+\sigma_{lv}$ we arrive at $h_{c}=2R_{c}$. In this limiting case, the work of critical cluster formation is equal to the value in the bulk, $\Delta G_{c}^{\left(cluster\right)}=\left(4\pi /3\right)R^{2}\sigma_{cl}$.

**Figure 6:**
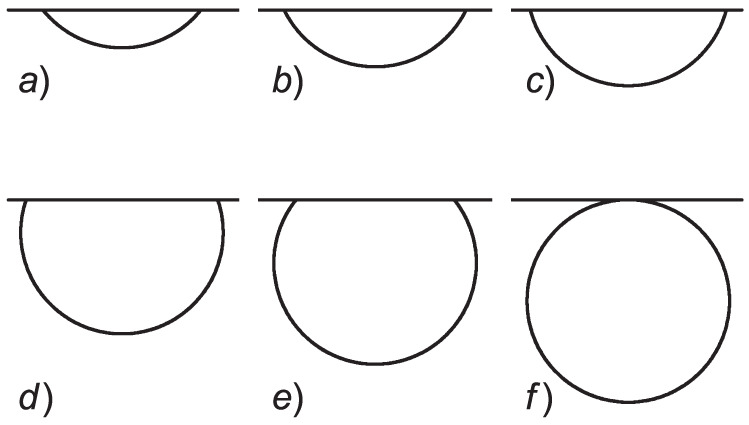
Possible shapes of the critical clusters in nucleation near planar interfaces in melting (top) and crystallization (bottom). (**a**–**f**) The different shapes result from different values of the ratio, $h_{c}/R_{c}$, as expressed by Equation (13) for melting and Equation (29) for crystal nucleation at planar interfaces. At the condition $\sigma_{cl}+\sigma_{lv}-\sigma_{cv}≅0$, shape (**a**) with limit $(h_{c}/R_{c})\to 0$ is realized in melting and shape (**f**) with limit $(h_{c}/R_{c})\to 2$ is realized in crystallization.

We come to the conclusion that crystal nucleation near interfaces proceeds preferentially at specified limiting conditions via the configuration shown in Figure 6f. An experimental example of this type of behavior was discussed by Avramov and Völksch in [49]. Their interpretation of this effect in that paper was different; here, we show that their results can be interpreted directly in terms of our developed model by accounting exclusively for surface tension effects.

## 3. Results and Discussion

In the present paper, the effect of planar interfaces on melting and crystallization is analyzed in terms of CNT. In line with the widely followed classical treatment of nucleation processes, bulk properties of the critical clusters are assumed to be the same as found for the newly evolving macroscopic phase and the capillarity approximation is employed. The analysis is performed for multi-component systems and is in this respect of general nature. The possible existence of heterogenous nucleation sites, specific features of the crystal structure or of the properties of the liquid near interfaces, the effect of elastic stresses, and other specific factors are not accounted for. In this way, we analyze the question of whether the mere existence of a liquid–vapor or crystal–vapor planar interface may affect the rate of nucleation processes in liquids or crystals. As is generally found in the application of CNT to nucleation, the results are expected at least to lead to a correct qualitative understanding of the peculiarities of nucleation near planar interfaces.

The results are expected to be similarly applicable to nucleation at curved interfaces, provided the curvature is not too high compared with the radii of the critical clusters. Additional effects may occur for rough interfaces, as discussed in connection with the analysis of surface structure on stress effects in crystal nucleation, e.g., in [9,10,50]. The specific features of nucleation near interfaces mentioned above and possible further factors may of course quantitatively modify the results, leaving, however, as we believe, the main conclusions unchanged. These main results can be summarized as follows.

Nucleation of liquids in the crystal at crystal–vapor planar interfaces proceeds as a rule with a much higher rate as compared to nucleation in the bulk of the crystal. Provided that the surface tensions of the liquid–crystal ($\sigma_{cl}$), liquid–vapor ($\sigma_{lv}$), and crystal–vapor ($\sigma_{cv}$) interfaces obey the condition $\sigma_{cv}=\sigma_{cl}+\sigma_{lv}$, the work of critical cluster formation tends to zero. In the range of values of the surface tension provided by the inequality $\sigma_{cv}<\sigma_{cl}+\sigma_{lv}$, this quantity is less than one half of the work of critical cluster formation for bulk nucleation. This result provides a direct interpretation of the effects of pre-melting discussed by A. Ubbelohde and cited in the introduction. In contrast, the existence of a liquid–vapor planar interface modifies the work of critical cluster formation in crystal nucleation of liquids to a much less significant degree. The work of critical crystal cluster formation is larger than one half of the bulk value of the work of critical cluster formation, reaching the limit at $\sigma_{cv}=\sigma_{cl}+\sigma_{lv}$. This result supports the theoretical concept that crystallization near interfaces is stimulated mainly by a reduction of the inhibiting effect of elastic stresses on crystal nucleation as compared to the bulk.

The shape of the critical clusters can be described in both considered cases by spherical caps with a radius $R_{c}$. The distance, $h_{c}$, from the cutting plane (coinciding with the crystal–vapor and liquid–vapor planar interfaces, respectively) to the top of the spherical cap representing the critical cluster varies for nucleation of a liquid in a crystal in the range $(h_{c}/R_{c})\leq 1$ and for crystal nucleation in a liquid in the range $2\geq (h_{c}/R_{c})\geq 1$. At $\sigma_{cv}=\sigma_{cl}+\sigma_{lv}$, the ratio $\left(h_{c}/R_{c}\right)$ of the critical cluster for nucleation in melting tends to zero. The critical crystallite has at this condition the shape of a sphere located tangentially to the liquid–vapor interface inside the liquid. These results are illustrated in Figure 6.

The results summarized above for nucleation in melting are obtained for the cases when the condition given by Equation (14) is fulfilled. In the theoretical analysis of wetting, a similar parameter, the spreading parameter, Ψ, is introduced, which can be expressed at certain conditions as [36]

(30) $$\Psi =\sigma_{cv}-\left(\sigma_{cl}+\sigma_{lv}\right)\;.$$

At Ψ<0, the liquid sticks to the surface and forms a cap. At Ψ>0, the liquid spreads, trying to cover the solid completely. The condition Ψ<0 corresponds to fulfillment of Equation (14), and the shapes of the critical clusters are similar to the shapes of the sessile drops formed in the kinetics of wetting processes. In the opposite case, perfect wetting has to be expected as the final result of the melting process, although the problem remains open of how it proceeds starting from a crystal–vapor planar interface.

The question arises here as to which shapes of critical clusters could be eventually suggested as leading to physically reasonable results if the condition given by Equation (14) does not hold. Alternatively, it is possible to assume that the properties of the critical clusters may significantly deviate from the properties in the bulk. In such cases, the surface tensions of the liquid critical cluster in contact with the crystalline solid $\left(\sigma_{cl}^{\left(cluster\right)}\right)$ and in contact with the vapor $\left(\sigma_{lv}^{\left(cluster\right)}\right)$ could be different from the values for planar interfaces. Such considerations are widely discussed in nucleation theory, starting with the work of Gibbs [32,40,51]. Gibbs also noted such a possibility as eventually being important in the description of nucleation of fluids in fluid–fluid interfaces. The values of the surface tensions for planar interfaces are shown here for the Lennard–Jones model system in Figure 4. Even when the inequality given by Equation (14) does not hold for planar interfaces, it may eventually be fulfilled for the surface tensions for the critical cluster. This topic we consider as an open problem which warrants further detailed research.

## Data Availability

Not applicable.
